# Reorganisation of the Basic Life Support Segment in a Physician-Led Emergency Medical Service: A Retrospective Evaluation of Operational and Clinical Characteristics

**DOI:** 10.3390/jcm15166364

**Published:** 2026-08-18

**Authors:** Julian Friebel, Marco Manni, Sophie-Charlott Gozdowsky, Paul Brettschneider, Delia Grün, André-Michael Baumann, Eiko Spielmann

**Affiliations:** 1 Emergency Medical Services Department, Berlin Fire and Rescue Service, Voltairestraße 2, 10179 Berlin, Germany; julian.friebel@berliner-feuerwehr.de (J.F.);; 2 Department of Dispatch Operations, Berlin Fire and Rescue Service, Voltairestraße 2, 10179 Berlin, Germany; 3 Medical Directorate of Emergency Medical Services Berlin, Berlin Fire and Rescue Service, Voltairestraße 2, 10179 Berlin, Germany

**Keywords:** emergency medical services (EMS), emergency medical dispatch, AMPDS, basic life support, advanced life support, dispatch stratification, low-acuity emergencies, resource allocation, patient safety, prehospital care

## Abstract

**Background:** Increasing demand for emergency medical services (EMS) challenges prehospital resource availability, particularly for time-critical emergencies requiring advanced life support (ALS). Although tiered BLS and ALS response models are established in many non-physician-led EMS systems, evidence regarding qualification-based dispatch stratification within physician-led EMS systems remains limited. This study evaluated the clinical and operational characteristics of an expanded BLS dispatch segment in Berlin EMS. **Methods:** A retrospective observational study analysed 2,132,246 EMS missions in Berlin, Germany, between 2020 and 2024. Missions were retrospectively classified according to dispatch codes included in the expanded BLS segment, designed to allocate incidents with lower expected requirements for ALS-level interventions to appropriately qualified EMS personnel. Analyses included dispatch characteristics, clinical findings from electronic patient care records, observed safety-related indicators, and ALS response intervals. **Results:** Overall, 28.7% of EMS missions were classified within the expanded BLS segment. Traumatic and psychiatric presentations represented the most frequent diagnostic groups. Based on predefined clinical indicators, no immediately life-threatening condition was documented in more than 95% of missions. Cardiopulmonary resuscitation occurred in 0.03% of cases, and emergency physician involvement was documented in approximately 3% of cases. **Conclusions:** A substantial proportion of EMS missions represented a population with predominantly lower expected prehospital treatment complexity within a qualification-based dispatch framework. Structured emergency call interrogation combined with dispatch classification and linked clinical data enabled retrospective evaluation of this approach. Further validation against independent clinical reference standards and linkage with downstream outcomes are required to determine broader applicability.

## 1. Introduction

Increasing demand for emergency medical services (EMS) has become a major challenge for healthcare systems worldwide. Rising emergency call volumes, workforce shortages, emergency department overcrowding and ambulance offload delays place growing pressure on prehospital resources and threaten the availability of advanced life support (ALS) capabilities for time-critical emergencies [1,2,3]. At the same time, a substantial proportion of EMS activations concern patients with lower-acuity presentations or situations with lower expected prehospital treatment complexity, highlighting the need for differentiated response strategies and appropriate allocation of limited prehospital resources [4].

Emergency medical dispatch represents the first point of resource allocation within the EMS chain and therefore plays a central role in balancing timely access to critical care with appropriate allocation of provider qualifications and resources. Standardised emergency call interrogation systems, such as the Advanced Medical Priority Dispatch System (AMPDS), are widely used internationally to support structured assessment and prioritisation of emergency calls [5]. However, dispatch decisions are inherently associated with uncertainty, as they rely on information available at the time of the emergency call rather than confirmed clinical diagnoses. Previous studies have therefore emphasised the importance of evaluating dispatch performance and understanding the relationship between dispatch information and subsequent clinical findings [6,7]. Linking dispatch information with routinely collected clinical EMS data may provide opportunities for continuous assessment and refinement of resource allocation strategies.

Qualification-based differentiation between BLS and ALS resources is established in many EMS systems, particularly in non-physician-led models. In physician-led EMS systems, however, emergency responses have traditionally relied more strongly on uniform deployment structures with limited differentiation according to provider qualification [8,9]. Increasing demand and resource constraints have stimulated interest in organisational approaches that allow available EMS resources to be allocated according to expected clinical requirements while maintaining access to advanced capabilities when needed [10,11,12,13,14].

In Berlin, ambulance deployments increased from 259,607 in 2004 to 513,318 in 2024, representing a 97% increase over two decades [15]. In response to these developments and increasing demands for prehospital resources, Berlin EMS introduced a reorganisation of the BLS segment in June 2023 [16]. The expanded segment enabled missions associated with selected dispatch codes with a lower expected need for advanced prehospital interventions to be managed by emergency medical technicians (Rettungssanitäter, RettSan) acting as lead clinicians, while maintaining appropriate response priorities and access to higher-qualified resources for incidents requiring advanced care. The approach was implemented within an established emergency medical dispatch framework and represented an additional pathway for matching provider qualification with expected prehospital care requirements.

The present study aimed to retrospectively evaluate the clinical characteristics and operational features associated with this expanded qualification-based BLS dispatch segment within a large physician-led EMS system. Specifically, we investigated the relationship between standardised dispatch-code allocation and observed clinical characteristics during EMS care, described observed safety-related indicators, and evaluated operational parameters related to ALS resource availability. The study was designed as an operational evaluation of an established EMS framework and not as an independent validation of dispatch accuracy or a causal assessment of system effects.

## 2. Materials and Methods

### 2.1. Study Design

This retrospective observational study evaluated the clinical characteristics and operational features associated with the expanded BLS segment within the Berlin EMS. The study assessed the distribution of dispatch codes included in the expanded BLS segment, associated clinical characteristics documented during EMS care, observed safety-related indicators, and operational parameters related to ALS resource availability.

**Figure 1:**
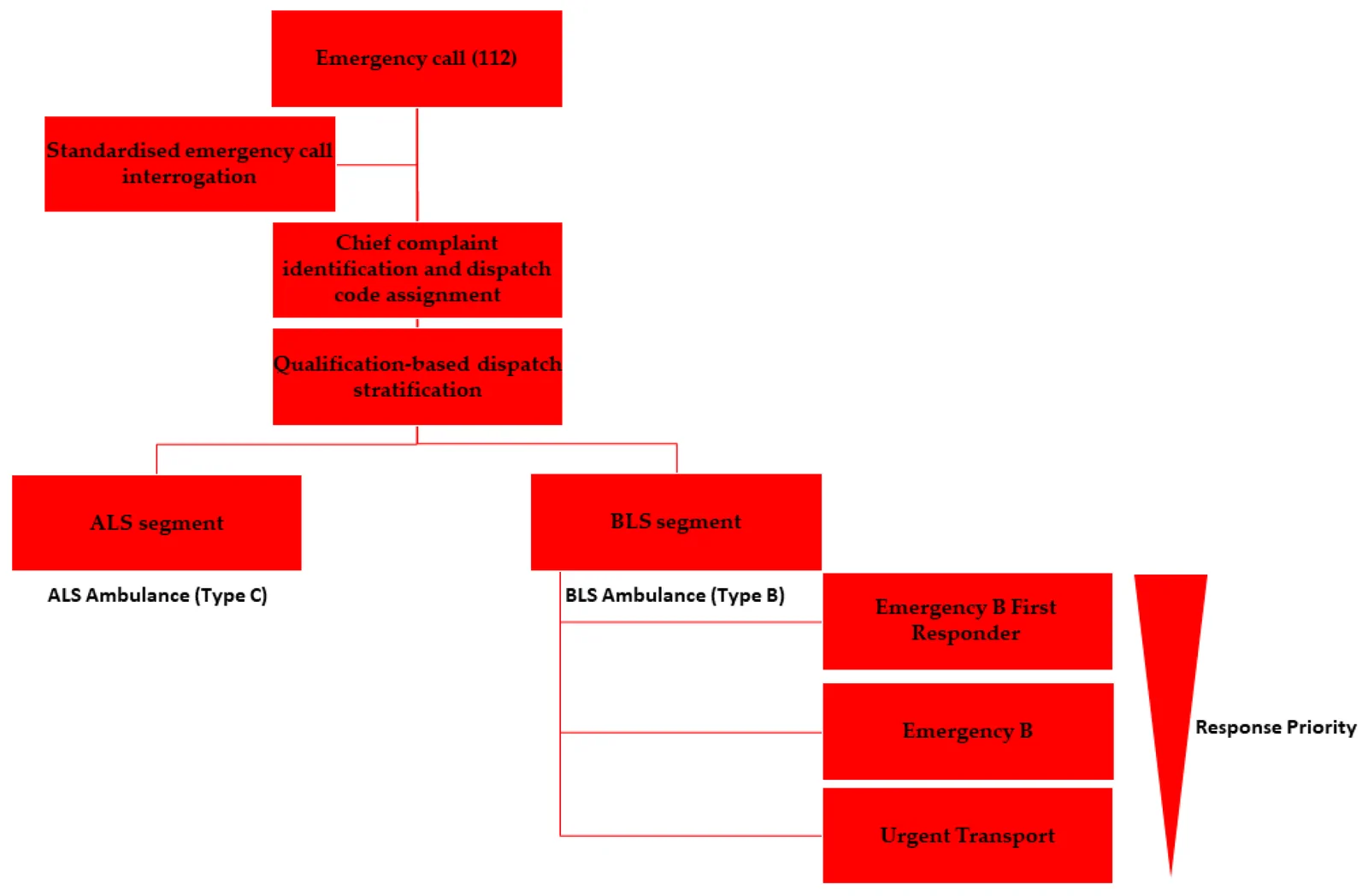
Qualification-based dispatch stratification within Berlin EMS. Operational response model after reorganisation and expansion of the BLS segment, integrating standardised emergency call interrogation, dispatch priorities, and provider qualification for differentiated BLS and ALS response pathways.

The expanded BLS segment represented a qualification-based dispatch framework in which selected emergency calls with a lower expected requirement for ALS-level interventions could be managed by BLS ambulances staffed by RettSan acting as lead clinicians. Stable operational use of the expanded BLS segment commenced in June 2023 (Figure 1).

**Figure 2:**
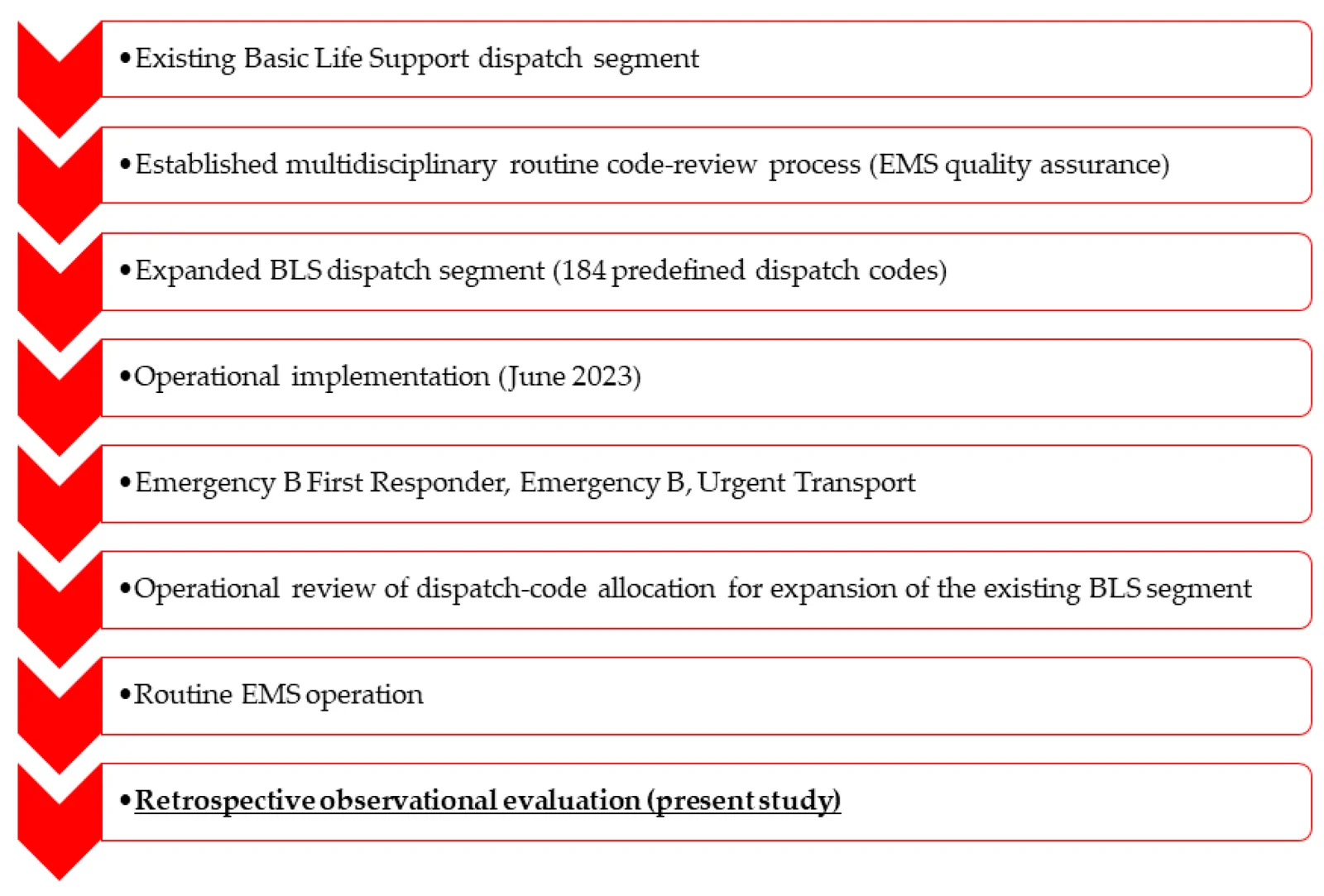
Expansion and retrospective evaluation of the existing BLS dispatch segment within the Berlin EMS. The figure illustrates the transition from the established BLS dispatch segment through the routine EMS quality management code-review process, operational expansion in June 2023, and subsequent retrospective evaluation in the present study. The expanded BLS segment comprised the three operational dispatch subgroups Emergency B, Emergency B First Responder, and Urgent Transport. The complete dispatch-code allocation analysed in this study is provided in Supplementary Table S1.

The present study was designed as an operational evaluation of an established EMS framework and was not intended as a validation study of dispatch accuracy or a causal assessment of effects attributable to expansion of the BLS segment (Figure 2).

### 2.2. Setting

Berlin EMS provides emergency medical care for approximately four million inhabitants, commuters, and tourists across an urban area of 891 km^2^. Emergency calls are managed centrally by the Berlin Fire and Rescue Service dispatch centre. Resource allocation is based on standardised emergency call interrogation using the Advanced Medical Priority Dispatch System^®^ (AMPDS) and Fire Priority Dispatch System^®^ (FPDS), which provide systemized assessment, prioritisation, and pre-arrival instructions for medical and fire-related emergencies (Figure 1).

Germany follows a physician-led Franco-German EMS model. Traditionally, transport-capable ambulances are staffed by paramedics (Notfallsanitäter, NotSan), who complete a three-year vocational training programme and provide advanced prehospital care. Emergency medical technicians (Rettungssanitäter, RettSan) undergo a three-month qualification programme. In Berlin, they may act as lead clinicians after additional two years of regular EMS practice or an equivalent of 2000 operational hours.

Prior to June 2023, most transport-capable ambulances operated within a predominantly ALS-oriented framework. Following an amendment of the Berlin Emergency Medical Services Act, selected incident groups with a lower expected requirement for ALS-level interventions became eligible for management by BLS ambulances staffed by RettSan personnel acting as lead clinicians [16].

Within the Berlin EMS structure, BLS and ALS ambulances share substantial operational and equipment characteristics, while differing primarily regarding the qualification profile of the responsible personnel. When additional physician-level expertise is required, emergency physician resources can be requested according to established EMS procedures.

Within the expanded BLS segment, three operational response categories were established according to dispatch priority and expected complexity (Figure 1):

- Emergency B First Responder: dispatch codes with potentially time-critical presentations, such as severe external bleeding or selected uncertain emergency situations. The dispatch system selects the resource with the shortest predicted arrival time, irrespective of whether this is a BLS or ALS ambulance. Additional first responder resources may be activated if required.
- Emergency B: urgent emergency calls for which the dispatch system primarily seeks a BLS ambulance within predefined response thresholds. These incidents are generally managed with emergency response priority using lights and sirens.
- Urgent Transport: lower-acuity incidents without immediate indication for emergency response, for which a BLS ambulance is requested within defined time limits without the use of lights and sirens. These cases include, among others, situations in which alternative outpatient pathways may not be feasible.

This qualification-based differentiation was designed to provide an additional response pathway for selected populations with lower expected requirements for ALS-level interventions while maintaining availability of higher-qualified resources for incidents with greater expected requirements for advanced prehospital care.

### 2.3. Establishment of the Expanded BLS Dispatch Framework

The expanded BLS dispatch framework was established through an existing emergency medical dispatch quality management process prior to the present study and was not initiated as part of this research project.

Within Berlin EMS, dispatch codes are routinely reviewed to ensure appropriate linkage between emergency call information, dispatch priorities, and required prehospital resources. Potentially suitable dispatch codes for inclusion in the expanded BLS segment were reviewed considering expected clinical severity, anticipated treatment requirements, provider competencies, and operational requirements. Furthermore, an age restriction was implemented to ensure ALS deployment for paediatric patients below 12 years of age. Therefore median age was higher after expansion of the BLS segment (Table 1).

The assessment focused on whether reported emergency situations were expected to be manageable by RettSan personnel acting as lead clinicians without an anticipated need for advanced interventions or emergency physician involvement.

Potentially appropriate dispatch codes were subsequently approved by the Chief Medical Director of EMS Berlin. The final operational allocation comprised 184 dispatch codes assigned to the expanded BLS segment (Supplementary Table S1). The currently implemented dispatch-code allocation is publicly available through the Berlin EMS Open Data platform.

For the present study, these predefined dispatch codes were retrospectively applied to EMS missions during the observation period to characterise associated clinical and operational features. The process therefore represents retrospective evaluation of an established dispatch framework rather than dispatch-code development or validation (Figure 2).

### 2.4. Data Sources and Study Population

Operational and clinical data were obtained from the electronic patient care record system (docYou med, pulsation^IT^) and the dispatch management system (IGNIS-Plus, Sopra Steria SE).

The primary study cohort comprised all EMS missions in Berlin, Germany, between 1 January 2020 and 13 September 2024, corresponding to 2,132,246 missions (Table 1). Inclusion was based on completed EMS missions for which a dispatch code had been generated following standardised emergency call interrogation and a response resource had been activated.

The unit of analysis was the individual EMS mission. Each mission represented a separate EMS encounter documented within the electronic patient care record system. The dataset did not contain patient-identifiable information enabling linkage of repeated EMS missions involving the same individual across different time points. Therefore, each EMS mission was analysed as an independent observation.

For retrospective classification, all EMS missions during the observation period were classified according to dispatch codes included in the expanded BLS segment. This classification was independent of the vehicle type subsequently dispatched and therefore does not indicate that a BLS ambulance was actually assigned or that the patient was exclusively managed by BLS personnel.

Importantly, individual dispatch codes were not linked to a single predefined response category in all situations. Operational response allocation could be modified based on contextual factors available during emergency call processing, including location, patient age, and specific dispatch-related considerations. Therefore, the same dispatch code could result in different response priorities or resource allocations depending on the operational context.

Due to technical requirements related to data extraction and processing of the large linked dispatch and clinical datasets, different analytical datasets were generated. The operational analyses included data up to 31 October 2024, whereas other analyses were based on the dataset available until 13 September 2024.

The observation period included a phase before and after operational introduction of the expanded BLS segment. These periods were analysed descriptively to characterise temporal changes in dispatch patterns, clinical findings, and operational parameters. The analysis was not designed as an interventional before-and-after evaluation.

To assess ALS resource availability, an additional analysis was performed using two identical three-month periods before and after introduction of the expanded BLS segment. Equivalent calendar periods were selected to minimise seasonal effects. The transition phase immediately following introduction was excluded from this comparison.

Electronic patient care documentation within the Berlin EMS system is subject to predefined documentation logic and plausibility checks during data entry. Extracted datasets undergo additional automated consistency checks during database processing. Before analysis, the extracted study dataset was manually reviewed for overall plausibility, including data structure, variable distributions, value ranges, and potential inconsistencies. Documentation completeness of relevant variables and handling of missing observations are described in Supplementary Table S2. Missing values were not imputed, and undocumented findings were not interpreted as normal findings. Analyses were performed using available documented information.

### 2.5. Data Linkage and Retrospective Dispatch-Code Classification

Because dispatch and clinical datasets were available within an integrated digital environment, dispatch information could be retrospectively linked with clinical findings documented during EMS care.

This enabled assessment of the relationship between predefined dispatch-code allocation and observed clinical characteristics, including physiological findings, suspected diagnoses, escalation requirements, and emergency physician involvement.

The linkage represented a retrospective evaluation of dispatch characteristics within routine EMS operations and was used to characterise the clinical profile of missions classified within the expanded BLS segment.

### 2.6. Study Measures

The study focused on clinical and operational characteristics associated with the expanded BLS segment.

Main study measures included:

- Proportion of EMS missions attributable to the expanded BLS segment;
- Distribution of dispatch codes and dispatch priorities;
- Suspected diagnoses documented by EMS personnel;
- Documented clinical findings;
- Emergency physician involvement;
- Observed cardiopulmonary resuscitation events;
- Critical physiological abnormalities;
- Hospital transport rates.

Additional operational measures included ALS ambulance response intervals measured from dispatch activation to arrival on scene.

### 2.7. Statistical Analysis

Continuous variables are presented as median and interquartile range (IQR), whereas categorical variables are expressed as absolute numbers and percentages.

Comparisons between groups were performed using the Mann–Whitney U test. A two-sided *p*-value < 0.05 was considered statistically significant.

Given the descriptive objective of the study and the large sample size, interpretation focused on the magnitude and clinical relevance of observed differences rather than statistical significance alone. Therefore, absolute differences and distributional characteristics were considered alongside statistical comparisons.

All analyses were performed using GraphPad Prism version 9.3.0 software.

### 2.8. Ethics

The study protocol was reviewed by the Ethics Committee of Charité—Universitätsmedizin Berlin, which raised no objections to the conduct of the study (reference number EA2/004/25).

## 3. Results

### 3.1. Dispatch Characteristics

**Figure 3:**
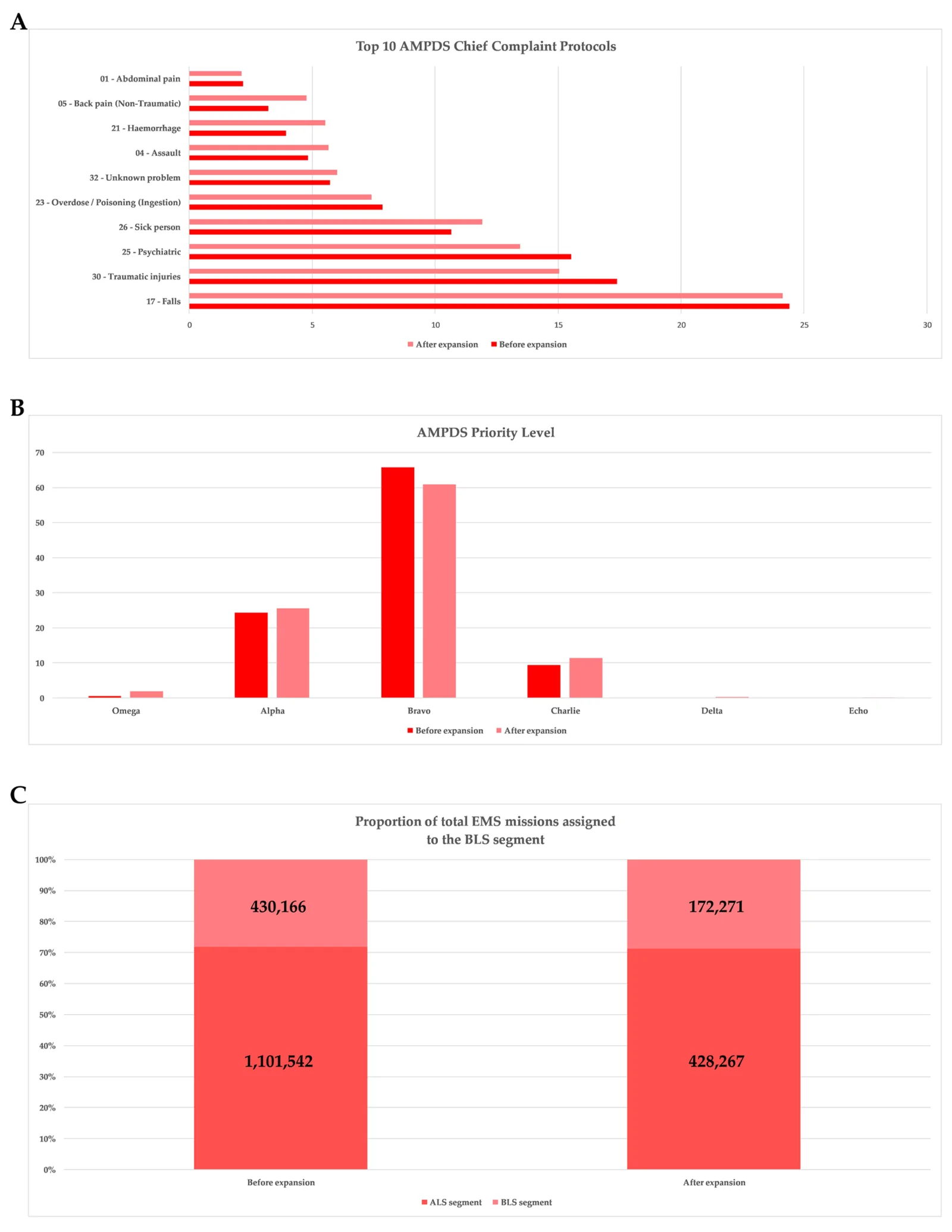
Dispatch characteristics and composition of missions classified within the expanded Basic Life Support segment. (**A**) Distribution of the ten most frequently used chief complaint protocols among missions classified according to predefined BLS dispatch codes, presented as percentages. (**B**) Distribution of dispatch priorities according to the Advanced Medical Priority Dispatch System^®^ (AMPDS) and Fire Priority Dispatch System^®^ (FPDS), presented as percentages. (**C**) Proportion of all EMS missions retrospectively classified according to the expanded BLS dispatch segment before and after expansion of the BLS dispatch code set, presented as percentages and absolute numbers.

Emergency call interrogation within the Berlin Fire and Rescue Service dispatch centre is based on standardised AMPDS and FPDS protocols, enabling structured prioritisation according to chief complaints, complexity and acuity categories (Figure 1). During the study period, minor traumatic presentations represented the largest group of missions retrospectively classified according to predefined BLS dispatch codes, followed by psychiatric emergencies (Figure 3A). The ten most frequently represented chief complaint protocols accounted for more than 95% of all missions within the expanded BLS segment.

Dispatch codes included in the expanded BLS segment consisted predominantly of a low-complexity population with respect to expected prehospital treatment requirements corresponding to AMPDS Alpha and Bravo categories (Figure 3B). To further differentiate operational response strategies, three response time subcategories were established within the expanded BLS segment: Emergency B First Responder (potentially time-critical emergency calls; 13.3%), Emergency B (urgent emergency calls; 69.5%), and Urgent Transport (lower-priority transport requests without immediate indication for emergency response; 17.2%) (Figure 1 and Figure 3B).

During the period before expansion of the BLS segment, 28.1% of all EMS missions were retrospectively attributable to the expanded BLS dispatch-code classification. During routine operation after expansion, this proportion remained comparable at 28.7% (Figure 3C), corresponding to approximately 375 missions per day.

Across both observation periods, 602,437 EMS missions fulfilled the predefined BLS dispatch-code criteria (430,166 before expansion and 172,271 after expansion). This classification was independent of the subsequently assigned EMS resource. Retrospective classification according to predefined BLS dispatch codes does not indicate that a BLS ambulance was actually dispatched. The subsequently assigned response resource depended on operational availability, predicted arrival times, and dispatch decisions. During routine operation after expansion, the proportion of missions classified within the BLS segment that were attended by a BLS ambulance increased from 24.12% to 62.31% (Table 1).

Across more than two million EMS missions, the expanded BLS dispatch segment represented a substantial and stable component of EMS workload and comprised a distinct population for qualification-based resource differentiation.

### 3.2. Clinical Characteristics and Observed Safety-Related Indicators

**Figure 4:**
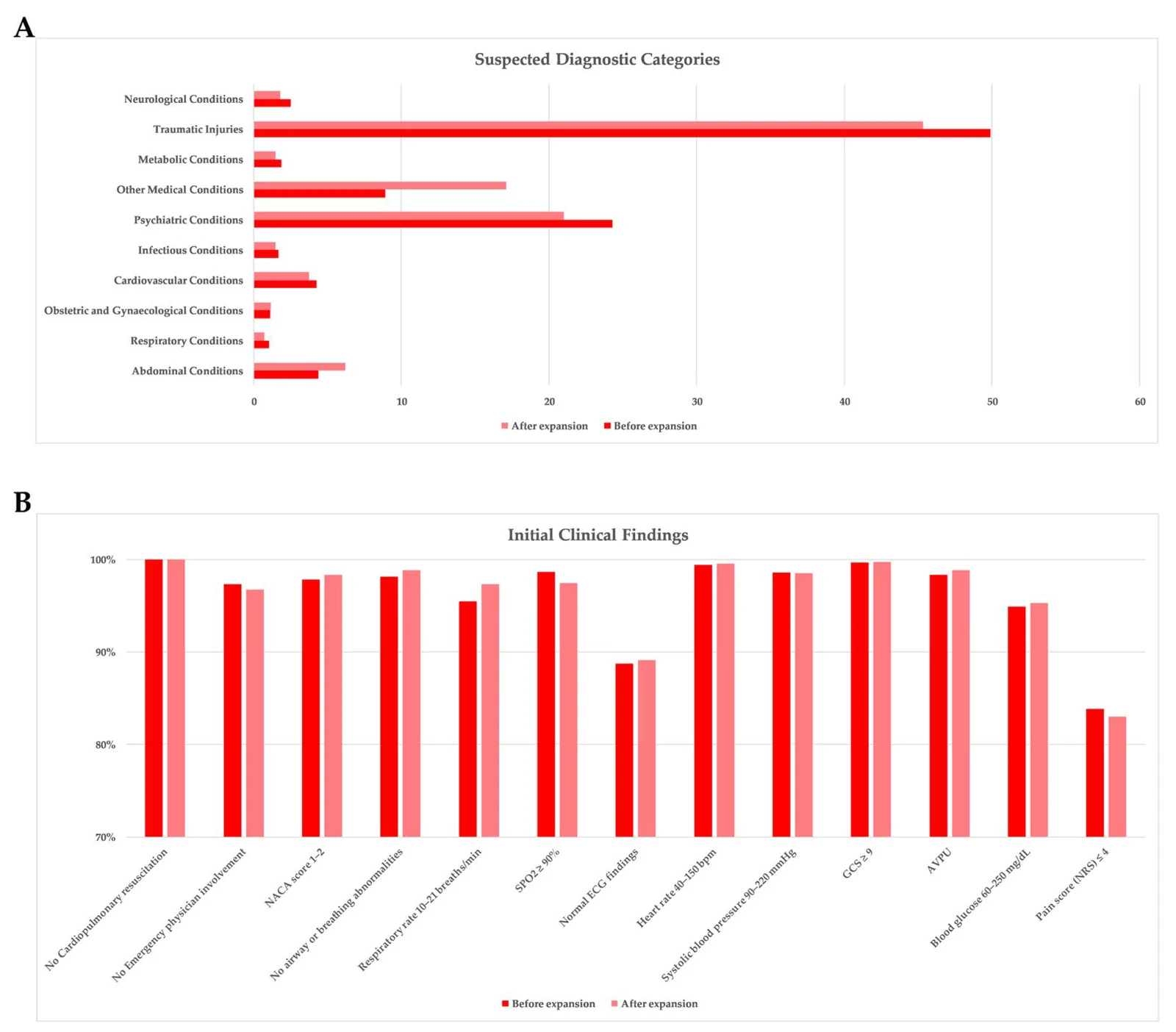
Clinical characteristics and observed critical findings among missions classified within the expanded Basic Life Support segment. (**A**) Relative distribution of suspected diagnostic categories documented by EMS personnel according to the German Minimum Emergency Dataset (MIND). (**B**) Initial clinical findings documented in electronic patient care records during the observation periods before and during routine operation after expansion of the BLS segment. Data are presented as percentages.

The expanded BLS segment was designed to include emergency call categories with a low expected probability of immediate advanced interventions and anticipated suitability for management by RettSan personnel acting as lead clinicians. Mild-to-moderate traumatic injuries and psychiatric disorders accounted for approximately two-thirds of suspected diagnoses documented by EMS personnel (Figure 4A).

Across both observation periods, out-of-hospital cardiac arrest requiring cardiopulmonary resuscitation was documented in 0.03% of missions classified within the expanded BLS segment. In absolute numbers, CPR-requiring cardiac arrest occurred in 129 missions before and 54 missions during routine operation of the expanded BLS segment.

Emergency physician involvement or a National Advisory Committee for Aeronautics (NACA) score greater than 2 was documented in fewer than 5% of missions. Emergency physician involvement occurred in 11,480 cases before (2.67%) and 5631 cases during routine operation after expansion (3.27%) (Figure 4B).

Airway abnormalities requiring intervention were documented in less than 2% of missions. Similarly, clinically relevant abnormalities of documented physiological parameters—including hypo- or hyperventilation, peripheral oxygen saturation below 90%, altered consciousness, Glasgow Coma Scale scores below 8, or hypo- and hyperglycaemia—were documented in fewer than 5% of missions (Figure 4B).

Normal electrocardiographic findings were documented in approximately 90% of missions with available ECG information. No, mild, or moderate pain (Numeric Rating Scale, NRS ≤ 4) was documented in 83% of missions with available pain assessment. Among documented medication administrations, balanced electrolyte solutions accounted for 56.96% and analgesics for 20.76%. No medication administration was documented in 98% of missions (Figure 4B).

Additional exploratory analyses included composite safety-related indicators stratified by chief complaint and dispatch code, CPR analyses according to chief complaint and dispatch code, and assessment of clinical deterioration parameters. Detailed results are provided in the Supplementary Material (Supplementary Tables S3–S7).

Overall, analysis of approximately 600,000 missions classified within the expanded BLS segment demonstrated a population predominantly characterised by lower expected requirements for advanced prehospital interventions, with infrequent observed critical findings. Immediately life-threatening findings were documented in a small proportion of missions. Hospital transport occurred in 75% of missions before and 78% during routine operation of the expanded BLS segment.

### 3.3. Operational Characteristics and ALS Resource Availability

The analysis of ALS response intervals was performed to assess operational parameters related to ALS resource availability after expansion of the BLS segment.

**Figure 5:**
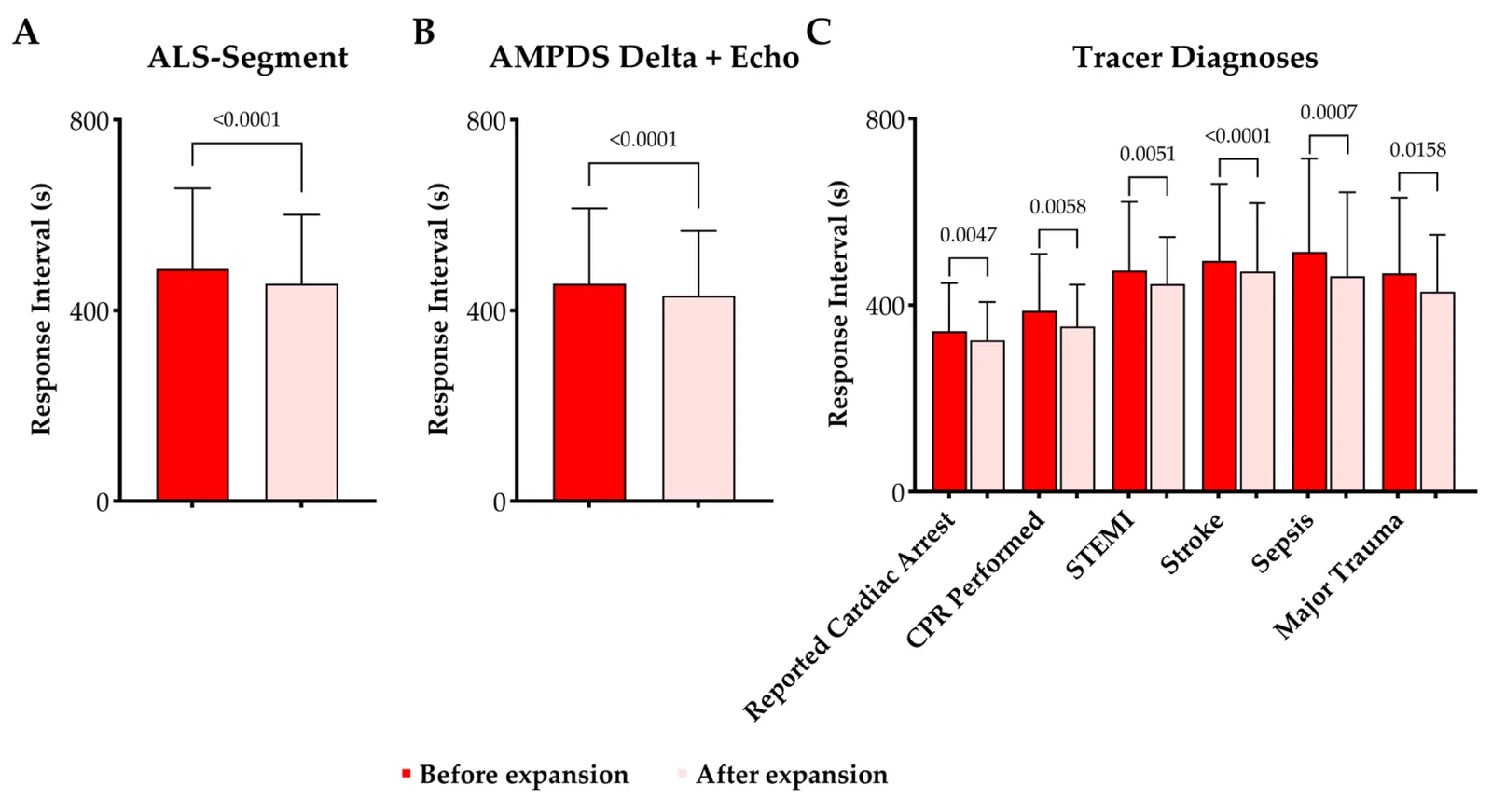
ALS ambulance response intervals before and after expansion of the BLS segment. Response intervals were measured from dispatch activation to arrival on scene. (**A**) All ALS missions. (**B**) High-priority missions corresponding to AMPDS Delta and Echo priorities. (**C**) Selected tracer conditions. Results are presented as median and interquartile range (IQR). Statistical comparisons were performed using the Mann–Whitney U test. Detailed response interval analyses stratified by tracer conditions are provided in Supplementary Table S8.

In the before-and-after comparison of predefined three-month periods, ALS ambulances reached ALS-priority incidents with a median reduction of 31 s in the interval from dispatch activation to arrival on scene (Figure 5A).

For high-priority missions classified as AMPDS Delta and Echo, median response intervals were reduced by approximately 25 s (Figure 5B).

Analysis of selected tracer diagnoses demonstrated reductions in median ALS response intervals ranging from 19 to 52 s during routine operation of the expanded BLS segment, corresponding to relative differences of approximately 5% to 10% (Figure 5C).

Detailed response intervals, including median values, interquartile ranges, sample sizes, absolute differences, relative differences, and statistical comparisons for tracer diagnoses, are provided in Supplementary Table S8.

During the observation periods, the proportion of missions retrospectively attributable to the expanded BLS segment remained stable despite increasing overall EMS workload. The observed operational findings are consistent with maintained ALS resource availability within the evaluated EMS system.

## 4. Discussion

In this study, we evaluated the clinical characteristics and operational implications of an expanded BLS dispatch segment within a physician-led urban EMS system. The evaluated framework was designed to allocate selected emergency calls with a lower expected requirement for ALS-level interventions to appropriately qualified non-physician EMS personnel, while maintaining access to ALS resources when required. In a large routine-care cohort, dispatch-code allocation was associated with a population predominantly characterised by lower expected requirements for advanced prehospital interventions, infrequent observed critical events, and preserved operational parameters related to ALS resource availability.

This study represents a retrospective operational evaluation of an established EMS strategy rather than a validation study of dispatch accuracy or a causal evaluation of the effects of BLS expansion. The findings should therefore be interpreted as describing the characteristics and operational consequences observed after expansion of a qualification-based dispatch framework under routine conditions. They do not demonstrate that the expanded BLS segment itself caused improvements in patient outcomes or EMS performance.

The clinical characteristics observed in this cohort were largely consistent with the intended target population of the expanded BLS segment. Mild-to-moderate traumatic presentations and psychiatric emergencies represented major diagnostic groups, whereas conditions requiring immediate advanced interventions were uncommon. These findings support the identification of a large EMS population with predominantly lower expected prehospital treatment complexity that may be suitable for differentiated resource allocation.

Importantly, allocation to the expanded BLS segment did not represent a strategy to avoid hospital transport or to classify these missions as non-medical encounters. Hospital transport remained frequent, occurring in 75% of missions before and 78% during routine operation of the expanded BLS segment. These findings emphasise that qualification-based differentiation primarily addresses the level of prehospital resources required rather than the necessity of medical evaluation or transport itself.

However, the observed clinical profile should not be interpreted as validation of dispatch accuracy. Medical dispatch systems aim to balance competing risks of undertriage and overtriage, and performance depends on multiple interacting factors, including caller information, protocol structure, local EMS organisation, and the definition of appropriate reference outcomes [17,18]. Recent evaluations of Medical Priority Dispatch System performance further emphasise the importance of context-specific monitoring and continuous quality assessment rather than assuming universal performance characteristics across EMS environments [19].

Accordingly, the relevance of the present approach does not lie in demonstrating perfect identification of all cases suitable for BLS-level management. Rather, the study illustrates the potential value of combining structured emergency call information with linked clinical EMS data to evaluate and continuously refine qualification-based resource allocation strategies.

### 4.1. Safety-Related Indicators

The present study included several complementary indicators to describe the observed clinical profile of missions allocated within the expanded BLS segment. These included emergency physician involvement, cardiopulmonary resuscitation events, clinical deterioration indicators based on available prehospital documentation, and additional routinely recorded clinical parameters.

These findings provide supportive information regarding the characteristics of the evaluated population but should be interpreted as observed safety-related indicators rather than evidence of definitive safety validation. In particular, the absence of frequent critical events within this large cohort supports the interpretation that the evaluated dispatch segment predominantly included missions with lower expected requirements for advanced prehospital interventions; however, it does not quantify undertriage, exclude individual cases of inappropriate allocation, or replace assessment against an independent clinical reference standard.

It is noteworthy that the highest proportions of CPR events were observed among nonspecific dispatch categories, reflecting the inherent uncertainty of emergency call information at the time of dispatch.

Emergency physician involvement requires careful interpretation in this context. Physician involvement represents an escalation pathway within EMS systems across a broad range of clinical situations and should not be considered a direct marker of inappropriate BLS allocation. Depending on local system organisation, physician involvement may represent appropriate escalation in a heterogeneous emergency population rather than failure of dispatch stratification. Furthermore, telemedical physician support structures were less established during parts of the observation period than in current practice, potentially influencing escalation patterns.

Future evaluation of dispatch safety will require linkage of dispatch and prehospital information with downstream clinical outcomes. Such linkage would enable assessment of patient-centred endpoints, delayed recognition of critical illness, hospital outcomes, and clinically meaningful measures of undertriage.

However, several implications for workforce development emerge from these findings. Clinical domains requiring specific assessment or management skills, such as pain management, electrocardiographic interpretation, and psychiatric emergencies, may represent areas in which additional competencies or support structures could further facilitate the implementation of differentiated response models. In this context, tele-emergency physician systems may represent valuable enablers by providing specialist support while preserving operational flexibility [20,21]. Rather than replacing conventional EMS structures, telemedicine may complement qualification-based dispatch models and provide escalation pathways when diagnostic uncertainty arises. Focused education in trauma assessment, psychiatric emergencies, digital documentation, and telemedical collaboration may further support the implementation of qualification-based response concepts.

### 4.2. Operational Implications and ALS Resource Availability

An important characteristic of the evaluated system is that dispatch allocation and vehicle deployment represent related but distinct processes. Not every incident assigned to the expanded BLS segment was necessarily attended by a BLS ambulance. Depending on operational availability, prioritisation, and resource distribution, ALS-capable resources remained available when required. This flexibility is a central element of the evaluated approach, as qualification-based dispatch differentiation aims to optimise resource allocation rather than rigidly assign every dispatch category to a single vehicle type.

The observed ALS response intervals provide additional information regarding system-level operational performance after expansion of the BLS segment. However, these findings require cautious interpretation. The analysis was not designed to determine whether changes in this magnitude translated into improved patient outcomes, and statistically significant differences in response intervals should not automatically be interpreted as clinically meaningful improvements.

The operational rationale for expanding the BLS segment was not primarily to achieve a predefined reduction in ALS response intervals, but to address increasing EMS demand and challenges regarding the availability of ALS-capable resources. In this context, maintaining operational performance despite increasing workload represents an important system-level objective. The observed changes in ALS response intervals should therefore be interpreted as descriptive indicators of resource allocation within the EMS system rather than evidence of improved individual patient outcomes.

Furthermore, the retrospective before-and-after design cannot exclude the influence of other concurrent developments, including changes in staffing, fleet capacity, dispatch processes, healthcare utilisation, or alternative care pathways. Although comparable observation periods were selected, causal attribution of observed operational changes to the BLS expansion is not possible.

Importantly, qualification-based dispatch differentiation does not primarily represent a prioritisation system. Rather, it aims to match the expected complexity of prehospital care with the required provider qualification, while maintaining appropriate response priorities for time-sensitive situations.

### 4.3. Relevance for Different EMS Models

Increasing demand for EMS has stimulated discussions regarding the appropriate management of lower-complexity emergency presentations and resource differentiation strategies. Importantly, increasing emergency number utilisation cannot be attributed solely to inappropriate low-acuity use of emergency services, and lower-complexity presentations represent a predictable component of modern EMS workload [22]. Consequently, strategies addressing this challenge should focus on appropriate allocation of available resources rather than simply reducing service utilisation. In the German EMS context, recent discussions have similarly highlighted the importance of professionalisation, qualification differentiation, and development of suitable care pathways for lower-priority emergency requests [23]. The evaluated BLS segment represents one possible organisational approach within this broader discussion.

The transferability of qualification-based dispatch differentiation requires consideration of local EMS structures. The specific Berlin model should not be interpreted as a universally applicable deployment concept. EMS systems differ substantially regarding workforce composition, physician involvement, dispatch structures, geography, transport distances, and available healthcare resources.

Nevertheless, the underlying principle may be relevant across different EMS models: structured emergency call information can support differentiation of available resources according to expected clinical requirements. In physician-led systems, such approaches may support preservation of physician-capable resources for patients with the highest expected need. In non-physician-led systems, similar principles may support prioritisation of advanced paramedic resources and appropriate allocation of available qualifications.

The relevance of such approaches is particularly dependent on geography and transport characteristics. An EMS mission considered suitable for BLS-level management in an urban environment with short transport distances may require different operational considerations in remote settings, where access times, environmental factors, and transport logistics substantially influence patient risk. Therefore, local adaptation rather than direct transfer of a specific model is essential.

## 5. Limitations

Several limitations should be considered. First, the retrospective observational before-and-after design does not allow causal conclusions regarding the effects of expanding the BLS segment. Observed changes may have been influenced by temporal trends, system-level developments, or other concurrent organisational changes.

Second, although the study evaluated a large urban EMS system, transferability to other settings depends on local dispatch structures, workforce models, geography, and healthcare organisation.

Third, the study was not designed as a validation study of dispatch accuracy. Sensitivity, specificity, undertriage, overtriage, and predictive performance of individual dispatch codes were not evaluated against an independent clinical reference standard.

Fourth, safety-related assessments were based on routinely documented prehospital information. Although documentation completeness was assessed, missing or undocumented findings could not be interpreted as normal findings. The available dataset did not allow assessment of hospital-based outcomes, long-term outcomes, or adverse events after EMS care.

Fifth, the unit of analysis was the EMS mission. As repeated EMS contacts by the same individual could not be identified, individual patients may have contributed more than one observation during the study period.

Furthermore, the expanded BLS segment was implemented for patients aged ≥12 years. This operational restriction reflects local considerations regarding provider training and implementation within the EMS system and may limit direct transferability to systems with different paediatric scopes of practice.

Finally, the study does not allow conclusions regarding the necessity of EMS utilisation or appropriateness of individual transport decisions.

## 6. Conclusions

Overall, this study provides a retrospective operational evaluation of an expanded qualification-based BLS dispatch segment within a structured physician-led EMS environment. The evaluated framework was associated with an EMS population characterised by lower expected prehospital treatment complexity across different response priority levels and with infrequent observed critical events, while ALS resource availability-related operational parameters were preserved.

The findings provide insights into the feasibility of qualification-based dispatch differentiation under routine conditions but should not be interpreted as validation of dispatch accuracy, proof of system safety, or evidence of causal effects on patient outcomes. Further studies incorporating independent clinical reference standards, external validation across different EMS environments, and linkage with downstream clinical outcomes are required to determine the broader applicability and clinical impact of this approach.

## Figures and Tables

**Table 1:** Characteristics of EMS missions and workload analysis periods before and after expansion of the BLS segment.

Characteristic	Before Expansion	After Expansion
Observation period	1 January 2020–10 June 2023	12 June 2023–13 September 2024
Duration	41 months	15 months
Total EMS activations	1,531,708	600,538
**BLS segment**		
Missions matching predefined BLS dispatch codes	430,166	172,271
Among these: attended by BLS ambulance	24.12%	62.31%
Among these: attended by ALS ambulance	75.88%	37.69%
**Mission-level demographic characteristics**		
Female	43.34%	44.23%
Male	56.44%	55.66%
Diverse	0.07%	0.11%
Undetermined	0.15%	0%
Age, years	38 (IQR 13–67)	43 (IQR 21–72)
**Operational workload analysis period**		
Observation period	1 August 2022–31 October 2022	1 August 2024–31 October 2024
Duration	3 months	3 months
Total EMS activations	110,651	114,942

Values are presented as percentages or median (interquartile range, IQR), as appropriate. Sex categories reflect routinely documented information. “Diverse” refers to documentation of a non-binary/other sex category. “Undetermined” indicates that the corresponding field was documented as unknown in the electronic record. EMS activations refer to completed EMS missions during the respective observation periods. Missions matching predefined BLS dispatch codes were identified retrospectively based on dispatch information and do not indicate that a BLS ambulance was assigned.

## Data Availability

Data are unavailable due to privacy restrictions.

## Supplementary Materials

The following supporting information can be downloaded at: https://www.mdpi.com/article/10.3390/jcm15166364/s1, Supplementary Table S1: Dispatch codes; Supplementary Table S2: Documentation completeness; Supplementary Table S3: Critical finding indicator by chief complaint; Supplementary Table S4: Critical finding indicator by dispatch code; Supplementary Table S5: CPR missions by chief complaint; Supplementary Table S6: CPR missions by dispatch code; Supplementary Table S7: Observed clinical changes during EMS care; Supplementary Table S8: ALS response intervals before and after BLS segment expansion.

## Abbreviations

The following abbreviations are used in this manuscript:

EMS	Emergency Medical Services
ALS	Advanced Life Support
BLS	Basic Life Support
RettSan	Emergency Medical Technician (Rettungssanitäter)
NotSan	Advanced Level Paramedic (Notfallsanitäter)
AMPDS	Advanced Medical Priority Dispatch System
FPDS	Fire Priority Dispatch System
NACA	National Advisory Committee for Aeronautics (NACA) score
CPR	cardiopulmonary resuscitation
MEES	Mainz Emergency Evaluation Score
NRS	Numeric Rating Scale
