# Supplementary material for: Reorganisation of the Basic Life Support Segment in a Physician-Led Emergency Medical Service: A Retrospective Evaluation of Operational and Clinical Characteristics
Source: J Clin Med. 2026 Aug 18;15(16):6364. doi: 10.3390/jcm15166364 (PMC13513053; doi:10.3390/jcm15166364)
Supplement: Supplementary file 1 [file jcm-15-06364-s001.zip › Supplementary_Material_Berlin_EMS.pdf]

Supplementary Material

Supplementary Table S1. Dispatch codes……………………………………………………………………...2

Supplementary Table S2. Documentation completeness……………………………………………….....3

Supplementary Table S3. Critical finding indicator by chief complaint…………………………………4

Supplementary Table S4. Critical finding indicator by dispatch code……………………………………5

Supplementary Table S5. CPR missions by chief complaint……………………………………………..6

Supplementary Table S6. CPR missions by dispatch code………………………………………………..7

Supplementary Table S7. Observed clinical changes during EMS care…………………………………...8

Supplementary Table S8. ALS response intervals before and after BLS segment expansion…………9

Supplementary Table S1. Dispatch codes assigned to the expanded Basic Life Support dispatch segment analysed in the present study. The table reflects the dispatch-code allocation used for retrospective classification of EMS missions within the expanded BLS segment.

This table is provided as a separate supplementary file due to its size.

Supplementary Table S2. Documentation completeness of clinical and operational variables during the observation periods before and after expansion of the BLS segment.

Variable	Before expansion of the BLS segment (%)	During routine operation after expansion of the BLS segment (%)
Sex information available	69.23	95.53
Age information available	78.14	100.00
MIND-based diagnostic group assignment available	70.60	95.84
CPR information available	100.00	100.00
Emergency physician involvement available	100.00	100.00
NACA score available	66.06	75.00
Airway/breathing assessment information available	66.51	95.38
ECG information available	10.76	10.66
Heart rate information available	51.01	78.52
Blood pressure information available	46.66	74.79
Level of consciousness information available	66.48	95.30
Pain score information available	61.23	86.09
Blood glucose information available	17.87	29.58
Medication administration information available	100.00	100.00
Transport mode information available	100.00	100.00
ALS ambulance response interval available	100.00	100.00

Percentages are calculated using all missions classified according to predefined BLS dispatch codes as denominator and indicate the proportion of missions for which the respective information was available in the analysed dataset. Missing information was not interpreted as normal findings.

Supplementary Table S3. Exploratory composite indicator of critical clinical findings by chief complaint.

Chief complaint	Composite n (%)	OR (95% CI)
07 – Burns / Explosion	198 (7,59 %)	2,83 (2,45–3,27)
05 – Back Pain	868 (4,96 %)	1,83 (1,70–1,96)
32 – Unknown Problem	1.272 (3,74 %)	1,35 (1,28–1,43)
13 – Diabetic Problems	168 (3,72 %)	1,32 (1,13–1,55)
30 – Traumatic Injuries	3.509 (3,49 %)	1,30 (1,25–1,35)
02 – Allergies / Envenomations	45 (3,40 %)	1,20 (0,89–1,62)
17 – Falls	4.581 (3,14 %)	1,15 (1,11–1,19)
04 – Assault / Sexual Assault	685 (2,21 %)	0,76 (0,71–0,82)
25 – Psychiatric / Abnormal Behaviour	1.966 (2,19 %)	0,74 (0,70–0,77)
26 – Sick Person	1.417 (2,14 %)	0,73 (0,69–0,77)
23 – Overdose / Poisoning	979 (2,11 %)	0,72 (0,68–0,77)
03 – Animal Bites / Attacks	25 (1,94 %)	0,68 (0,45–1,00)
01 – Abdominal Pain	361 (1,89 %)	0,65 (0,59–0,72)
21 – Haemorrhage / Lacerations	449 (1,79 %)	0,61 (0,56–0,68)
24 – Pregnancy / Childbirth	34 (0,87 %)	0,30 (0,21–0,42)
18 – Headache	32 (0,84 %)	0,29 (0,20–0,41)
16 – Eye Problems	37 (0,74 %)	0,25 (0,18–0,35)
20 – Heat / Cold Exposure	5 (0,45 %)	0,16 (0,06–0,37)

Only chief complaints with at least 500 missions classified according to predefined BLS dispatch codes were included. The composite indicator comprised emergency physician involvement and/or cardiopulmonary resuscitation. Missing information was not interpreted as absence of abnormal findings.

Supplementary Table S4. Composite critical finding indicator by dispatch code.

Dispatch Code	Composite n (%)	OR (95% CI)
07A01	181 (7,97 %)	2,98 (2,56–3,47)
17B04A	86 (6,77 %)	2,49 (2,00–3,10)
25B03B	43 (5,95 %)	2,16 (1,59–2,95)
32B03	435 (5,24 %)	1,92 (1,74–2,11)
17B03P	68 (4,99 %)	1,80 (1,41–2,30)
05C00	862 (4,95 %)	1,82 (1,70–1,96)
17B04	590 (4,58 %)	1,66 (1,53–1,81)
04B02A	74 (4,52 %)	1,62 (1,28–2,05)
17A02P	441 (4,34 %)	1,56 (1,42–1,72)
30A02	1.797 (3,71 %)	1,36 (1,29–1,42)
13C00	166 (3,71 %)	1,32 (1,13–1,54)
17A02	1.063 (3,70 %)	1,33 (1,25–1,42)
17B04P	212 (3,62 %)	1,29 (1,12–1,48)
30B00	141 (3,62 %)	1,29 (1,09–1,52)
30B01	1.367 (3,60 %)	1,30 (1,23–1,38)
32B04	354 (3,56 %)	1,27 (1,14–1,41)
17B03	78 (3,37 %)	1,19 (0,95–1,50)
02B00	42 (3,34 %)	1,18 (0,87–1,61)
17B00	215 (3,34 %)	1,19 (1,03–1,36)
23C07I	225 (3,27 %)	1,16 (1,01–1,32)

The table presents the 20 dispatch codes with the highest proportion of missions with emergency physician involvement and/or cardiopulmonary resuscitation among dispatch codes with at least 500 missions classified within the expanded BLS dispatch segment. Odds ratios were calculated by comparing each dispatch code with all remaining missions classified within the expanded BLS dispatch segment. Analyses included all 602,437 missions classified within the expanded BLS segment. Odds ratios represent exploratory measures of association and should not be interpreted as causal effects or measures of dispatch accuracy.

Supplementary Table S5. Distribution of cardiopulmonary resuscitation missions by chief complaint within the expanded BLS dispatch segment.

Chief complaint	Missions with CPR performed, n (%)	Proportion of all CPR missions (%)	OR (95% CI)
32 – Unknown Problem	74 (0.217%)	40.4	11.36 (8.46–15.27)
26 – Sick Person	22 (0.033%)	12.0	1.11 (0.71–1.73)
17 – Falls	41 (0.028%)	22.4	0.90 (0.64–1.28)
23 – Overdose/Poisoning	11 (0.024%)	6.0	0.77 (0.42–1.41)
25 – Psychiatric / Abnormal Behaviour	9 (0.010%)	4.9	0.30 (0.15–0.58)
30 – Traumatic Injuries	9 (0.009%)	4.9	0.26 (0.13–0.50)

Odds ratios (ORs) were calculated by comparing each chief complaint with all remaining missions classified within the expanded BLS dispatch segment. To reduce unstable estimates due to sparse data, only protocols with at least five CPR events are shown. These analyses describe the distribution of observed CPR events among retrospectively classified EMS missions and do not represent an assessment of dispatch sensitivity or undertriage.

Supplementary Table S6. Distribution of cardiopulmonary resuscitation missions by dispatch code within the expanded BLS dispatch segment.

Dispatch code and Berlin dispatch text	Missions with CPR performed, n (%)	Proportion of all CPR missions (%)	OR (95% CI)
32B03 Unclear emergency situation	40 (0.482%)	21.9	20.13 (14.17–28.60)
32B04 Unclear emergency situation; caller speaking a foreign language could not be understood	21 (0.211%)	11.5	7.74 (4.91–12.20)
26B01 Unknown condition in a request for medical assistance	12 (0.189%)	6.6	6.59 (3.67–11.83)
17B04P Unknown condition following a fall in a public place	9 (0.154%)	4.9	5.28 (2.70–10.32)
17B04 Unknown condition following a fall	18 (0.140%)	9.8	5.00 (3.07–8.13)
32B01 Unclear emergency situation	9 (0.061%)	4.9	2.05 (1.05–4.01)
23B01I Overdose without emergency warning signs	6 (0.038%)	3.3	1.27 (0.56–2.87)
30B01 Injury	7 (0.018%)	3.8	0.59 (0.28–1.26)
26B00 Unknown condition in a request for medical assistance	9 (0.018%)	4.9	0.56 (0.28–1.09)

Distribution of all cardiopulmonary resuscitation (CPR) events according to dispatch code. Odds ratios (ORs) were calculated by comparing each dispatch code with all remaining missions classified within the expanded BLS dispatch segment. Only dispatch codes fulfilling both criteria (≥5 CPR events and ≥500 total missions) are shown. The denominator for CPR event distribution comprised all missions classified within the expanded BLS dispatch segment (n = 602,437). These analyses describe the distribution of observed CPR events among retrospectively classified EMS missions and do not represent an assessment of dispatch sensitivity or undertriage.

Supplementary Table S7. Observed changes in clinical parameters during EMS care among missions classified within the expanded BLS segment.

Delta MEES, median change [IQR]	+1 [0-2]
Decrease in Glasgow Coma Scale (GCS) to ≤8	0.22%
Decrease in oxygen saturation (SpO_2) below 90%	6.76%
Decrease in systolic blood pressure below 90 mmHg	2.41%
ECG rhythm change to a more severe clinically relevant category	0.19%
New onset of airway compromise or need for ventilatory support during EMS care	0.02%

Observed clinical change indicators were assessed among missions classified according to the expanded BLS dispatch segment. Percentages refer to the proportion of documented cases with available information for the respective parameter. These indicators represent observed clinical findings during EMS care and should not be interpreted as validation of dispatch accuracy or definitive proof of safety.

Supplementary Table S8. ALS ambulance response intervals before and after expansion of the BLS segment.

Response category / tracer condition/ tracer conditions	number	Before expansion of the BLS segment, median [IQR], s	During routine operation after expansion of the BLS segment, median [IQR], s	Absolute difference, s	Relative difference, %	p-value
All ALS missions	193.126	487 [360-656]	456 [342-601]	-31	-6.37	<0.0001
AMPDS Delta and Echo	50.199	456 [339-614]	431 [325-567]	-25	-5.48	<0.0001
Reported cardiac arrest	1.395	344 [262-447]	325 [252-407]	-19	-5.52	0.0047
CPR Performed	549	388 [290-510]	354 [272-444]	-34	-8.76	0.0058
STEMI	654	474,5 [375-621]	445,5 [336-621]	-29	-6.11	0.0051
Stroke	5.341	495 [370-660]	472 [357-619]	-23	-4.65	<0.0001
Sepsis	793	514 [393-714]	462 [343-642]	-52	-10.12	0.0007
Major Trauma	230	468 [347-631]	429 [292-551]	-39	-8.33	0.0158

ALS ambulance response intervals were measured from dispatch activation to arrival on scene. Comparisons were performed using predefined three-month observation periods before and after expansion of the BLS segment. Values are presented as median (IQR).
